# Multilocus Variable-Number Tandem-Repeat Analysis of Yersinia ruckeri Confirms the Existence of Host Specificity, Geographic Endemism, and Anthropogenic Dissemination of Virulent Clones

**DOI:** 10.1128/AEM.00730-18

**Published:** 2018-08-01

**Authors:** Snorre Gulla, Andrew C. Barnes, Timothy J. Welch, Jesús L. Romalde, David Ryder, Michael J. Ormsby, Jeremy Carson, Karin Lagesen, David W. Verner-Jeffreys, Robert L. Davies, Duncan J. Colquhoun

**Affiliations:** a Norwegian Veterinary Institute, Oslo, Norway; b University of Queensland, Brisbane, Australia; c USDA-ARS-NCCCWA, Leetown, West Virginia, USA; d Departamento de Microbiología y Parasitología, CIBUS-Facultad de Biología, Universidade de Santiago de Compostela, Santiago de Compostela, Spain; e Cefas, Weymouth Laboratory, Weymouth, England; f University of Glasgow, Glasgow, Scotland; g Department of Primary Industries, Parks, Water and Environment, Hobart, Tasmania, Australia; h University of Bergen, Bergen, Norway; University of Illinois at Urbana-Champaign

**Keywords:** Atlantic salmon, MLST, MLVA, Yersinia ruckeri, fish pathogen, geographic endemism, host specificity, molecular typing, rainbow trout, yersiniosis

## Abstract

A multilocus variable-number tandem-repeat analysis (MLVA) assay was developed for epizootiological study of the internationally significant fish pathogen Yersinia ruckeri, which causes yersiniosis in salmonids. The assay involves amplification of 10 variable-number tandem-repeat (VNTR) loci in two five-plex PCRs, followed by capillary electrophoresis. A collection of 484 Y. ruckeri isolates, originating from various biological sources and collected from four continents over 7 decades, was analyzed. Minimum-spanning-tree cluster analysis of MLVA profiles separated the studied population into nine major clonal complexes and a number of minor clusters and singletons. The major clonal complexes could be associated with host species, geographic origin, and serotype. A single large clonal complex of serotype O1 isolates dominating the yersiniosis situation in international rainbow trout farming suggests anthropogenic spread of this clone, possibly related to transport of fish. Moreover, subclustering within this clonal complex indicates putative transmission routes and multiple biotype shift events. In contrast to the situation in rainbow trout, Y. ruckeri strains associated with disease in Atlantic salmon appear as more or less geographically isolated clonal complexes. A single complex of serotype O1 exclusive to Norway was found to be responsible for almost all major yersiniosis outbreaks in modern Norwegian salmon farming, and site-specific subclustering further indicates persistent colonization of freshwater farms in Norway. Identification of genetically diverse Y. ruckeri isolates from clinically healthy fish and environmental sources also suggests the widespread existence of less-virulent or avirulent strains.

**IMPORTANCE** This comprehensive population study substantially improves our understanding of the epizootiological history and nature of an internationally important fish-pathogenic bacterium. The MLVA assay developed and presented represents a high-resolution typing tool particularly well suited for Yersinia ruckeri infection tracing, selection of strains for vaccine inclusion, and risk assessment. The ability of the assay to separate isolates into geographically linked and/or possibly host-specific clusters reflects its potential utility for maintenance of national biosecurity. The MLVA is internationally applicable and robust, and it provides clear, unambiguous, and easily interpreted results. Typing is reasonably inexpensive, with a moderate technological requirement, and may be completed from a harvested colony within a single working day. As the resulting MLVA profiles are readily portable, any Y. ruckeri strain may rapidly be placed in a global epizootiological context.

## Abstract (precis)

This comprehensive population study substantially improves our understanding of the epizootiological history and nature of an internationally important fish-pathogenic bacterium. The MLVA assay developed and presented represents a high-resolution typing tool particularly well suited for Yersinia ruckeri infection tracing, selection of strains for vaccine inclusion, and risk assessment. The ability of the assay to separate isolates into geographically linked and/or possibly host-specific clusters reflects its potential utility for maintenance of national biosecurity. The MLVA is internationally applicable and robust, and it provides clear, unambiguous, and easily interpreted results. Typing is reasonably inexpensive, with a moderate technological requirement, and may be completed from a harvested colony within a single working day. As the resulting MLVA profiles are readily portable, any Y. ruckeri strain may rapidly be placed in a global epizootiological context.

## INTRODUCTION

Yersinia ruckeri, a member of the family Enterobacteriaceae, causes the systemic infection yersiniosis, commonly known as enteric redmouth disease (ERM), predominantly in salmonid fish. The bacterium has a global distribution and is found in all countries where salmonids are presently cultured. Internationally and economically, yersiniosis is most commonly associated with farmed rainbow trout, although in a few countries, including Norway, Australia, Scotland, and Chile, significant numbers of outbreaks in farmed Atlantic salmon occur (1–4). In Norway, the incidence of yersiniosis in Atlantic salmon has increased considerably in recent years, and the disease is currently a major concern to the Norwegian aquaculture industry. While transmission of the bacterium is believed to occur primarily in freshwater, disease outbreaks in Atlantic salmon are also seen following sea transfer and, to an increasing degree in Norway, in larger sea-farmed fish. It is suspected that outbreaks at sea may be related to stress-induced activation of subclinical infections.

Previous studies have identified a considerable degree of inter-strain variation in biochemical (5) and outer membrane protein (6, 7) profiles within the Y. ruckeri species. Two biotypes have been described: biotype 1 (motile, phospholipase secreting) and biotype 2 (nonmotile, non-phospholipase secreting) (5). Biotype 2 strains have evolved independently in several different continents, and it has been hypothesized that evolution of biotype 2 from biotype 1 has been driven by large-scale vaccination against biotype 1 strains in rainbow trout (2, 8–10). Different serotyping systems have been described for Y. ruckeri (11–13), which has resulted in a rather complex and somewhat confusing serologically based nomenclature, comprehensively reviewed by Barnes in 2011 (14). Recently, a new serotype (O8) was identified as the most commonly isolated serotype from Atlantic salmon in recent years in Scotland (7).

There are a relatively limited number and range of genetic studies on Y. ruckeri (14). Multilocus sequence typing (MLST) (15, 16) has, however, identified 39 different sequence types among strains isolated from several different fish species, a single mammal (muskrat), and the environment. A pulsed-field gel electrophoresis assay affording relatively high resolution has also been developed (8), but this technique is labor-demanding, and the results may not always be readily comparable between laboratories. Recent whole-genome sequence (WGS) analysis of a number of Y. ruckeri isolates, recovered largely in Tasmania, Australia, has shed light on some of the evolutionary processes at work within this species (2). There is no doubt that WGS combined with bioinformatics analysis offers the highest degree of resolution of all typing systems. Both WGS and advanced bioinformatics capabilities remain restricted, however, to a relatively small number of laboratories. To our knowledge, no established molecular epizootiological typing system capable of rapid and unambiguous identification and separation of isolates at the sub-MLST level exists for Y. ruckeri.

Multilocus variable-number tandem-repeat analysis (MLVA), based on identification of variable-number tandem-repeat (VNTR) DNA sequences at a number of loci in bacterial genomes, affords highly transportable data, is fast and inexpensive, and offers strain resolution in some cases almost matching that of WGS (17–19). MLVA is now accepted as a reference typing method for many bacterial species and has been utilized in typing of several fish-pathogenic species, including, among others, Francisella noatunensis (20), Edwardsiella piscicida (21), and Renibacterium salmoninarum (22).

As Y. ruckeri is an important fish pathogen internationally and of increasing significance in Norwegian aquaculture, there is an acute need for development of a rapid and affordable molecular typing tool capable of sub-MLST resolution. The aim of the present study was, therefore, to establish an MLVA assay for Y. ruckeri.

## RESULTS

### MLVA development and deployment.

Following identification and verification of putative VNTR regions in the Y. ruckeri genome, 10 informative loci were selected for inclusion in the present MLVA and divided equally among two multiplex PCR assays (Table 1). Capillary electrophoresis (CE) performed on PCR products detected peaks corresponding to all 10 VNTR loci in 480 of the 484 Y. ruckeri isolates examined, with the four remaining isolates apparently lacking 1 to 3 loci (see Tables S1 and S2 in the supplemental material). Amplicons were easily distinguished based on fluorescent labeling and size (see Fig. S1 in the supplemental material). Within each of the two respective multiplex assays developed, no overlap in size was observed between identically labeled loci. Split peaks in electropherograms separated by a single base pair, a common CE artifact due to incomplete nontemplated 3′ adenosine addition, were occasionally observed despite an extended final extension period (60 min). In such cases, the longer fragment was consistently selected for downstream analysis.

**TABLE 1:** Observed characteristics of each VNTR locus^*a*^

VNTR locus	Multiplex assay	Repeat sequence	PCR fragment size range (bp)	Repeat count	No. of unique alleles	Simpson's index of diversity
YR2365^*b*^	I	GCCAGAA	195–475	7–47	32	0.87
YR3168	I	TATTCTC	101–319	1–32	29	0.80
YR1524^*c*^	I	TGAGGTAT	391–519	2–18	14	0.67
YR2276^*c*^	I	AATCC	123–243	4–28	18	0.71
YR3750^*b*^	I	ATGGCGTA	339–635	3–40	25	0.84
YR1070	II	ATATCCT	198–437	4–38	27	0.83
YR57^*c*^	II	CACTGC	94–160	2–13	12	0.83
YR940	II	TTTAGTGG	313–585	1–35	30	0.86
YR1899^*b*^	II	CCTGATAAA	105–222	2–15	13	0.84
YR2794^*b*^^,^^*c*^	II	CATGAC	443–509	4–15	9	0.64

a PCR fragment size ranges and repeat counts were calculated from corrected capillary electrophoresis fragment size calls (see Materials and Methods and Results).

b PCR fragment size ranges, repeat counts, and unique allele counts exclude cases of missing amplicons in this locus.

c Minor repeat sequence heterogeneity was observed in this locus.

Due to the predictable nature of discrepancies identified between PCR fragment size as called by CE and Sanger sequencing, locus-specific correctional values were calculated (see Fig. S2 in the supplemental material) and employed for improved precision of CE-based VNTR fragment size calling. Single-base-pair deletions identified in a few strains in the downstream flank of VNTR loci YR3168 and YR1070 did not affect the number of predicted repeats. A total of 329 unique MLVA profiles were detected among the 484 Y. ruckeri isolates typed.

### Allelic diversity and statistical evaluation.

The allelic diversity within individual VNTR loci varied between 9 and 32 alleles (not counting missing amplicons), with Simpson's index of diversity (SID) values ranging from 0.64 to 0.87 (Table 1). The SID for all 10 loci combined was >0.99, indicating the very high probability of separating nonclonal isolates. LIAN analysis resulted in a standardized index of association (*I*_A_^s^) of 0.2772, which differs significantly from zero (*P*_Monte Carlo_ < 0.0001). This confirms linkage disequilibrium, reflecting a low rate of recombination and the clonal nature of the investigated population.

### MLVA cluster analysis.

**FIG 1:**
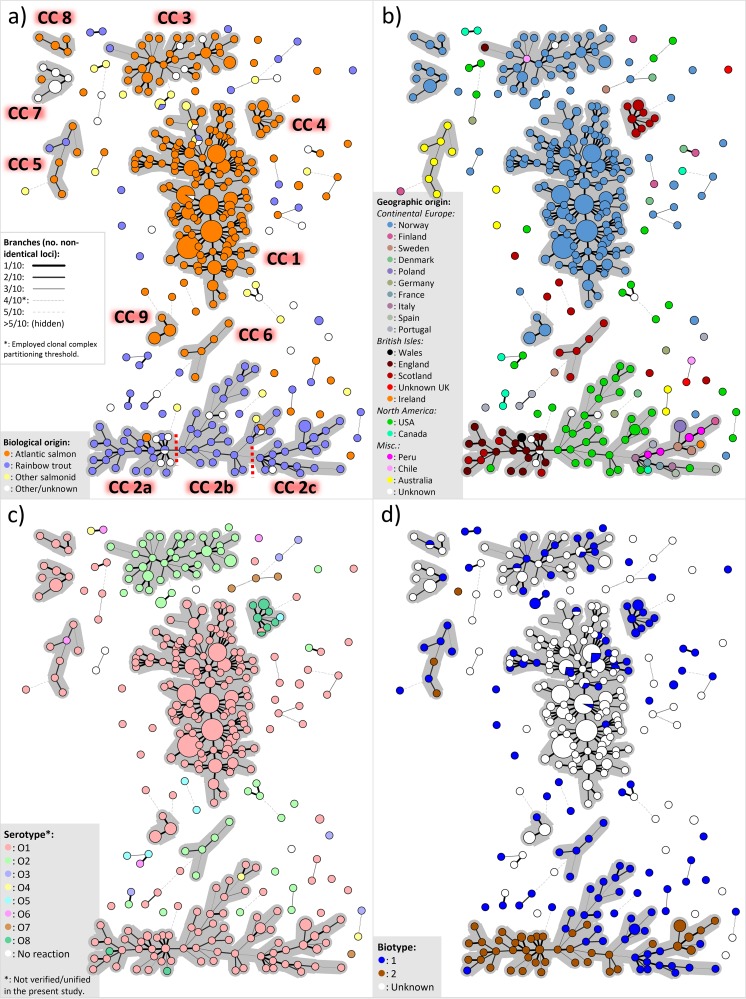
Minimum-spanning trees based on MLVA data from 484 Y. ruckeri isolates (see Table S2 in the supplemental material). The four diagrams are topographically identical but are colored according to different metadata, i.e., biological origin/host (a), geographic origin (b), serotype (c), and biotype (d); details are given in the bottom left of each panel. Branch representations for declining MLVA similarity and clonal complex (CC) annotations (with CC 2 subdivisions marked by dotted red lines) are shown in panel a.

Minimum-spanning-tree (MST) cluster analysis of MLVA profiles, utilizing a relatively stringent cluster partitioning threshold (≤4/10 nonidentical loci), placed 83% of the studied isolates within either of nine major clonal complexes (CC), each comprising five or more isolates. These clonal complexes were strongly biased toward one or more epizootiological attributes, e.g., host fish species, geographic origin, and/or serotype (Fig. 1 and Tables 2 and S1). The remaining isolates represented either singletons or minor clusters. While extensive allele variation was evident in all 10 loci (Table 1), all 484 studied isolates could be linked to at least one other isolate by three or more common VNTR alleles.

**TABLE 2:** Major MLVA clonal complexes with incorporated strains/isolates and associated metadata trends^*a*^

MLVA clonal complex	Associated strains/isolates	Isolate count	MLVA profile count	Main serotype(s)^*b*^ (%)	Biotype(s) (%)	Main biological origin (%)	Geographic origin(s) (%)	Temporal span	MLST sequence type(s)^*c*^
1	NVI-1182, NVI-1292, NVI-1311, NVI-1313, NVI-1318, NVI-1319, NVI-1369, NVI-2365, NVI-2577, NVI-3629, NVI-3737, NVI-3829, NVI-3868, NVI-4006, NVI-4007, NVI-4047, NVI-4118, NVI-4124, NVI-4132, NVI-4148, NVI-4185, NVI-4256, NVI-4335, NVI-4564, NVI-4570, NVI-4841, NVI-4854, NVI-5233, NVI-5305, NVI-5570, NVI-5599, NVI-5824, NVI-5847, NVI-5858, NVI-5942, NVI-6061, NVI-6092, NVI-6130, NVI-6274, NVI-6282, NVI-6287, NVI-6288, NVI-6347, NVI-6348, NVI-6351, NVI-6362, NVI-6393, NVI-6414, NVI-6415, NVI-6526, NVI-6614, NVI-6615, NVI-6616, NVI-6617, NVI-6618, NVI-6619, NVI-6620, NVI-6621, NVI-6678, NVI-6732, NVI-6919, NVI-6939, NVI-6940, NVI-6950, NVI-7013, NVI-7059, NVI-7108, NVI-7142, NVI-7146, NVI-7212, NVI-7231, NVI-7306, NVI-7348, NVI-7349, NVI-7470, NVI-7485, NVI-7512, NVI-7514, NVI-7525, NVI-7531, NVI-7542, NVI-7632, NVI-7934, NVI-7935, NVI-7968, NVI-8066, NVI-8074, NVI-8076, NVI-8202, NVI-8387, NVI-8421, NVI-8422, NVI-8507, NVI-8508, NVI-8522, NVI-8524, NVI-8525, NVI-8526, NVI-8527, NVI-8539, NVI-8559, NVI-8567, NVI-8618, NVI-8668, NVI-8670, NVI-8680, NVI-8749, NVI-8898, NVI-9018, NVI-9021, NVI-9055, NVI-9082, NVI-9162, NVI-9163, NVI-9240, NVI-9241, NVI-9336, NVI-9394, NVI-9395, NVI-9396, NVI-9397, NVI-9587, NVI-9588, NVI-9589, NVI-9590, NVI-9592, NVI-9593, NVI-9594, NVI-9595, NVI-9596, NVI-9597, NVI-9598, NVI-9654, NVI-9656, NVI-9657, NVI-9698, NVI-9700, NVI-9706, NVI-9730, NVI-9731, NVI-9732, NVI-9808, NVI-9809, NVI-9810, NVI-9811, NVI-9812, NVI-9813, NVI-9814, NVI-9818, NVI-9844, NVI-9902, NVI-9915, NVI-9916, NVI-9949, NVI-9967, NVI-10025, NVI-10026, NVI-10049, NVI-10050, NVI-10051, NVI-10052, NVI-10055, NVI-10084, NVI-10085, NVI-10127, NVI-10197, NVI-10208, NVI-10215, NVI-10216, NVI-10217, NVI-10234, NVI-10252, NVI-10254, NVI-10358, NVI-10361, NVI-10394, NVI-10401, NVI-10403, NVI-10428, NVI-10429, NVI-10470, NVI-10499, NVI-10512, NVI-10515, NVI-10541, NVI-10542, NVI-10561, NVI-10577, NVI-10592, NVI-10600, NVI-10622, NVI-10704, NVI-10705, NVI-10706, NVI-10717, NVI-10724, NVI-10806, NVI-10860, NVI-10935, NVI-10936, NVI-10946, NVI-10951, NVI-10974, NVI-10975, NVI-10976, NVI-10977, NVI-10978, NVI-10979, NVI-10981, NVI-10982, NVI-10985, NVI-10986, NVI-10987, NVI-10988, NVI-10989, NVI-10990, NVI-11020, NVI-11021, NVI-11022, NVI-11023, NVI-11024, NVI-11025, NVI-11026, NVI-11027, NVI-11028, NVI-11036, NVI-11087, JR-1533, NVH_3758	229	112	O1 (100)	1 (100)	S. salar (97)	Norway (100)	1986–2017	3
2									
a	NVI-1185, NVI-1265, DVJ-04025, DVJ-84015, DVJ-84016, DVJ-85051, DVJ-86020, DVJ-86021, DVJ-86027, DVJ-86038, DVJ-86043, DVJ-86052, DVJ-93010, DVJ-93046, DVJ-99086, DVJ-99167, NVH_3754, NVH_3756, NVH_3759, RD330, RD388, RD516, RD518, RD520, RD538, RD556, TW-F186	27	25	O1 (93)	1 (4), 2 (96)	O. mykiss (91)	UK (96), USA (4)	1984–2013	1
b	ATCC 29473^T^, CCM6094, CSF007-82, JR-11.73, NCTC12266, RD386, TW-11.1, TW-11.26, TW-11.28, TW-11.33, TW-11.34, TW-11.4, TW-11.40, TW-11.5, TW-11.54, TW-11.55, TW-11.68, TW-11.69, TW-11.70, TW-F182, TW-F184, TW-F185, TW-F190, TW-F191, TW-F196, TW-F198, TW-F199, TW-F200, TW-F201, TW-HEA-280, TW-HEA-302	31	29	O1 (97)	1 (68), 2 (32)	O. mykiss (90)	USA (100)	1961–2016	1, 29
c	NVI-1316, NVI-1317, NVI-1381, NVI-1382, NVI-1383, NVI-1384, NVI-1385, NVI-9925, NVI-10996, NVI-10997, NVI-10998, NVI-10999, NVI-11000, NVI-11002, NVI-11004, NVI-11007, JR-1, JR-11, JR-15, JR-32, JR-9, JR-CA10, JR-RS75, TW-F183, TW-F195	25	19	O1 (100)	1 (48), 2 (52)	O. mykiss (96)	Europe (68), Peru (20), N. America (12)	1978–2017	1, 9, 10, 11, 24, 28
3	NVI-1177, NVI-1178, NVI-1291, NVI-1347, NVI-1366, NVI-1367, NVI-1594, NVI-1660, NVI-2274, NVI-2329, NVI-2953, NVI-2954, NVI-4365, NVI-5621, NVI-6225, NVI-6833, NVI-7400, NVI-7970, NVI-8331, NVI-8363, NVI-8510, NVI-8511, NVI-8512, NVI-8513, NVI-8710, NVI-9327, NVI-9681, NVI-9815, NVI-9968, NVI-10199, NVI-10290, NVI-10404, NVI-10517, NVI-10937, NVI-10980, NVI-10983, NVI-10984, DVJ-86047, JR-6807, NVH_3755	40	32	O2 (100)	1 (100)	S. salar (97)	Norway (94), England (3), Chile (3)	1986–2016	8, 40, 42
4	RD340, RD404, RD426, RD446, RD468, RD502, RD512, RD530, RD558, RD564, RD570, RD576	12	8	O8 (83)	1 (100)	S. salar (100)	Scotland (100)	2002–2014	3
5	QMA0397, QMA0427, QMA0431, QMA0435, QMA0440, TW-11.43, TW-11.44	7	7	O1 (86)	1 (71), 2 (29)	S. salar (71)	Australia (100)	1959–2014	44, 47
6	RD336, RD444, RD514, RD542, RD552	5	5	O2 (100)	1 (100)	S. salar (100)	Scotland (100)	2001–2013	NA^*f*^
7	NVI-4098, NVI-11074, NVI-11075, NVI-11076, NVI-11077, NVI-11078, NVI-11079, NVI-11080, NVI-11081, NVI-11082, NVI-11083	11	6	O1 (100)	1 (100)	Biofilm (91)^*d*^	Norway (100)	1999–2017	NA
8	NVI-10587, NVI-10588, NVI-10590, NVI-10591, NVI-11055	5	4	O1 (100)	1 (100)	S. salar^*e*^ (100)	Norway (100)	2015–2017	43
9	NVI-11049, NVI-11050, NVI-11053, NVI-11054, NVI-11056, NVI-11057, NVI-11058, NVI-11059, NVI-11060	9	3	O1 (100)	1 (100)	S. salar^*e*^ (100)	Norway (100)	2017	NA
Minor clonal complexes and singletons	NVI-343, NVI-488, NVI-495, NVI-499, NVI-500, NVI-1290, NVI-1365, NVI-1386, NVI-1387, NVI-1389, NVI-1398, NVI-1399, NVI-2135, NVI-2197, NVI-2205, NVI-2275, NVI-2328, NVI-2775, NVI-2909, NVI-2966, NVI-2970, NVI-3779, NVI-4479, NVI-4493, NVI-4507, NVI-4840, NVI-4987, NVI-5089, NVI-5635, NVI-8270, NVI-9924, NVI-10589, NVI-11065, NVI-11073, JR-11.29, JR-11.47, JR-2/85, JR-2599, JR-820317, JR-830118, JR-850812, JR-860821, JR-B14-102, JR-B16-8, JR-B9-28, JR-BV216, JR-C10-19, JR-E842, JR-RS2, JR-RS54, JR-RS80, NCTC12267, NCTC12268, NCTC12269, NCTC12270, NVH_3757, QMA0401, QMA0424, QMA0436, QMA0438, RD154, RD338, RD356, RD358, RD370, RD406, RD544, TW-11.30, TW-11.31, TW-11.46, TW-11.49, TW-11.50, TW-11.51, TW-11.57, TW-11.59, TW-11.62, TW-11.65, TW-11.66, TW-11.76, TW-11.97, TW-15-041, TW-15-066, TW-16-050	83	79	O1 (51), O2 (23)	1 (98), 2 (2)	Salmonids (90)	Europe (65), North America (29), Australia (5), Chile (1)	1974–2017	3, 4, 5, 6, 7, 13, 15, 17, 18, 19, 20, 21, 22, 23, 25, 27, 33, 34, 35, 40, 41, 45, 46

a See Fig. 1 for major MLVA clonal complexes. Details for individual strains/isolates are provided in Table S1 in the supplemental material. Isolates for which a particular trait was not known were disregarded when assessing the distribution of that trait.

b Serotype affiliations of individual strains as previously assigned; they were not verified/unified in the present study.

c MLST sequence types following some modifications to the initial scheme (see Results).

d Ten of 11 isolates in CC 7 were recovered from biofilm in a single freshwater salmon farm (no clinical symptoms were or had been reported).

e All isolates in CC 8 and 9 were recovered from the egg fluid of clinically healthy brood stock in a single salmon farm.

f NA, not available.

### VNTR stability.

**FIG 2:**
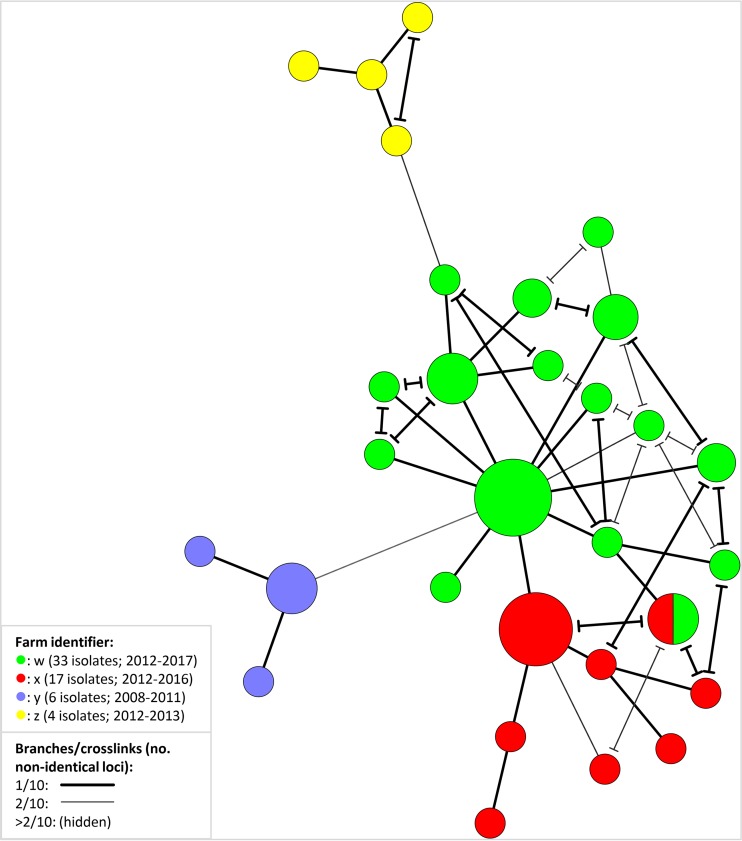
Minimum-spanning tree based on MLVA of clonal complex 1 (see Fig. 1) isolates associated with four Atlantic salmon smolt farms (w, x, y, and z; see details in the figure) in Norway which had experienced recurrent yersiniosis outbreaks. Cross-links showing all possible connections involving ≤2/10 nonidentical VNTR loci are shown (see details in the figure).

Four of the six Y. ruckeri strains (representing various clonal complexes) typed following 0, 10, 20, 30, and 40 culture passages revealed no changes within the 10 VNTR loci. A single strain acquired one additional repeat copy in YR1899 between passages 0 and 10 and subsequently two more copies in this locus between passages 30 and 40, while another single strain acquired one additional repeat copy in YR1070 between passages 30 and 40. MLVA typing of 19 Y. ruckeri isolates recovered from 13 fish during a single yersiniosis outbreak in a commercial salmon farm revealed a single extra repeat copy in YR3750 in one isolate compared to the remaining 18. Separate MST analysis involving four collections of epizootiologically related isolates verified farm-specific subclustering within CC 1 (Fig. 2).

### Comparative resolution of MLST versus MLVA.

Sequence inconsistencies were discovered in two loci (*thrA* and *recA*) for identical strains in two previously published MLST studies (15, 16). The sequence differences, which occurred throughout the respective data sets, were situated in the 6 to 13 terminal base pairs (both termini) of *thrA* and in the final base pair of *recA* (see Fig. S3 in the supplemental material). BLAST searches of available Y. ruckeri whole-genome sequences consistently identified sequences in agreement with the latest study (16). To obtain uniformity, the ambiguous sequence termini were removed prior to MLST meta-analysis in the current study, resulting in locus sizes of 286 and 471 bp for *thrA* and *recA*, respectively, versus 303 to 305 bp (*thrA*) and 472 bp (*recA*) in previous publications (15, 16).

These changes resulted in integration of the previously published *thrA* allele types (AT) 2, 5, and 6 into *thrA* AT 1, 1, and 4, respectively, thereby making *thrA* AT 2, 5, and 6 obsolete. Similarly, *recA* AT 5 was integrated into *recA* AT 1, making *recA* AT 5 obsolete. Consequently, sequence types (ST) 2, 12, 14, 26, 31, 32, 38, and 39 (15, 16) were rendered obsolete, and the strains involved were integrated into ST 1, 9, 7, 18, 1, 15, 22, and 3, respectively (see Table S3 in the supplemental material). One novel allele type in each of the loci *glnA* (AT 11), *dnaJ* (AT 8), *thrA* (AT 7), and Y-HSP60 (AT 6) were identified during the current study, and corresponding sequences were submitted to NCBI GenBank. Eight novel sequence types (ST 40 to 47) were identified. Notably, no sequence could be identified for the *glnA* locus (AT set to 0) within the PacBio-generated genome of strain QMA0440 (ST 47), and a 12-bp insertion was identified in *dnaJ* in strain QMA0436 (ST 46).

Comparison of MST diagrams based on MLST and MLVA data from 134 Y. ruckeri isolates revealed largely consistent clustering patterns and verified the considerably higher resolution of MLVA, with the 35 MLST sequence types included discriminated further into 123 distinct MLVA profiles (see Fig. S4 in the supplemental material). Multilocus sequence analysis (MLSA) based on concatenation of the truncated housekeeping gene sequences further highlighted the phylogenetic distances between the investigated MLST sequence types (see Fig. S5 in the supplemental material).

## DISCUSSION

Infectious bacterial diseases of humans, plants, and animals are commonly caused by the emergence and spread of host-adapted, virulent endemic clones (see, e.g., references 23, 24, and 25). Characterization of clinical isolates to the clonal and subclonal levels is, therefore, essential to better understand the underlying epizootiology of any particular disease toward development of avoidance strategies and to aid selection of relevant strains for vaccine development. MLVA has been successfully deployed to describe the epidemiology/epizootiology at various scales for a number of bacterial pathogens of plants (26), mammals (27), and fish (20–22). We have developed a ten-locus MLVA assay for the fish pathogen Yersinia ruckeri and employed it to characterize the population structure within a collection of 484 isolates derived from highly diverse spatiotemporal and biological origins. Our findings support the previous contention (2) that this bacterium has an almost pan-global endemic distribution comprising, with the exception of some anthropogenically transported strains, geographically distinct and host-specific populations.

The MLVA is robust and internationally applicable, as proven by the detection of all 10 VNTR loci in >99% of the examined isolates. The minor variability following serial *in vitro* passage and low intra-outbreak variability, together with a combined SID of >0.99, suggest that the assay combines levels of both stability and variability suitable for epizootiological use. Largely consistent minimum-spanning-tree (MST) clustering of MLST and MLVA data underpins the suitability of both methods for inferring the Y. ruckeri population structure. The considerably higher level of strain differentiation provided by MLVA relative to MLST illustrates, however, the greater utility of MLVA for epizootiological study of “local” strains of the same MLST sequence type (see Fig. S4 in the supplemental material). MLST, which relies on sequence variability in evolutionarily conserved genes, may well provide a more unified picture of the overall population structure, simply due to its lower resolution.

Internationally, yersiniosis is most economically important in rainbow trout farming, and disease in this fish species is most commonly associated with Y. ruckeri serotype O1 strains of both biotypes 1 and 2. MLVA allocated 83% of the 81 serotype O1 isolates from rainbow trout examined to a single large clonal complex (CC 2), with the remaining isolates primarily occupying minor clonal complexes or appearing as singletons (Fig. 1a). CC 2 could be further subdivided into three main subpopulations (denoted a, b, and c), of which CC 2a and CC 2b comprise isolates originating almost exclusively from the United Kingdom and the United States, respectively, whereas CC 2c contains isolates primarily from continental Europe but also from Ireland, North America, and Peru (Fig. 1b). The presence of three geographically biased subpopulations within CC 2, with the “central” positioning of the U.S. cluster, is consistent with a situation in which CC 2 strains have spread from North America on at least two separate occasions: once to the United Kingdom and once to continental Europe and/or South America. The presumed direction of spread is further supported by the fact that the first detection of CC 2b (United States) predates that of the two other CC 2 subpopulations by 17 years or more. Due to the proximity within CC 2c of the relatively recent Peruvian isolates (recovered in 2008) to those from continental Europe, it appears likely that they both descend from the same North American lineage. Combined, these findings are supportive of the reported existence of geographically confined subpopulations of rainbow trout-associated Y. ruckeri serotype O1 in the mentioned regions (8).

Nonmotile Y. ruckeri biotype 2 strains increasingly dominate the disease situation in rainbow trout farming in many countries, reflecting independent and parallel evolution, presumably provoked by vaccines targeting flagellar antigens (8, 9, 28). In this regard, the proportion of rainbow trout isolates classified in the present study as biotype 2 increases from 33% to 77% for those recovered before and after the turn of the century, respectively. Clonal expansion of these mutants in the post-vaccination era is visualized in Fig. 1d, in which groups of biotype 2 isolates from rainbow trout form defined sublineages within CC 2. In the case of the geographically widely distributed CC 2c, MLVA clustering indicates emergence of biotype 2 in continental Europe after introduction of biotype 1 from North America (9) and is also consistent with an independent biotype shift in South America. This is in contrast to the biotype 2 phenotype of the United Kingdom-associated CC 2a, which appears to have been introduced from North America as biotype 2 (9). If this is in fact the case, however, it is hard to explain the occurrence of a single biotype 1 isolate, recovered in England 9 years after the first detection of CC 2a in the country, relatively deep within this clonal complex, unless it represents reversion from biotype 2 to biotype 1.

Two Scottish rainbow trout isolates described as serotype O8, a serotype most commonly associated with Atlantic salmon (7), were found within CC 2a (Fig. 1c), a situation which could be explained by recombinational events involving the lipopolysaccharide (LPS) biosynthesis cascade. The importance of LPS has recently been verified in elicitation of a protective immune response against Y. ruckeri in rainbow trout (29), and, conceivably, vaccine-related evolutionary pressures similar to those associated with the independent emergences of the various Y. ruckeri biotype 2 lineages may have prompted this putative serotype shift.

Yersiniosis in farmed Atlantic salmon is a significant problem in Norway, Scotland, Australia (Tasmania), and Chile and may be associated with various Y. ruckeri serotypes, although serotype O1 is generally considered to be the most virulent (2, 4, 7). In contrast to the situation with rainbow trout, most Y. ruckeri isolates from Atlantic salmon were separated by MLVA into discrete (unlinked) clonal complexes specific to particular geographic regions (Fig. 1a and b and Table 2). The clonal complex currently dominating in Australian salmon farming (CC 5) includes two isolates recovered in 1959 in Victoria, Australia. This predates the import of Atlantic salmon to Tasmania between 1984 and 1986 from a landlocked population in New South Wales (established with Canadian stock in 1965) and therefore verifies the native status of this clone in Australia (2). Geographically biased clustering was also identified among isolates from Norwegian (CC 1 and 3) (see below) and Scottish (CC 4 and 6) Atlantic salmon (Table 2). The high degree of diversity and spatially linked clustering among clinical Y. ruckeri isolates from Atlantic salmon supports, therefore, the previously proposed geographic endemism in this bacterium (2, 7, 8). Unfortunately, too few isolates from Chilean and North American salmon were examined to corroborate the existence/absence of salmon-specific clones in these areas.

While Y. ruckeri serotype O2 is sporadically detected in Norway, almost all major yersiniosis outbreaks in modern Norwegian salmon farming have been associated with serotype O1. Knowledge of the genetic diversity of Norwegian strains is scarce, however, and very few isolates had been characterized prior to the present study. Of the 286 isolates examined from Atlantic salmon in Norway, recovered between 1985 and 2017, we found 77% to belong to CC 1 (exclusively serotype O1) and 12% to CC 3 (exclusively serotype O2). Although CC 1 contains only Norwegian isolates, this clone may share a relatively recent ancestry with the “Scottish” CC 4, as they belong to the same MLST sequence type, in addition to three VNTR loci being entirely conserved across both of these clonal complexes. This association could conceivably be explained by geographic proximity, as may the presence of a single English isolate peripherally in the predominantly “Norwegian” CC 3 (Fig. 1b). In contrast, the appearance of a Chilean isolate (from 2008) relatively deep within this clonal complex is more likely to reflect anthropogenic spread. Despite the evident existence of various serotype O1 clones in Norway (Fig. 1b and c and Table 2), all serotype O1 isolates recovered from clinical yersiniosis cases in Norwegian Atlantic salmon since 1995 to date belong to CC 1, indicating the relatively high virulence of this clonal complex toward this fish species. MLVA clustering of isolates associated with individual freshwater farms over several years further verifies persistent colonization of these farms by individual CC 1 strains (Fig. 2).

As with most previous investigations involving Y. ruckeri, the collection examined in the present study is dominated by clinical isolates from diseased fish. As such, the scrutinized material provides a poor basis for investigation of the genetic structure within the overall population of what may well be an essentially environmental bacterial species. The disproportionate frequency of isolation of certain genotypes from diseased fish against a background of other genotypes does, however, provide support for increased host specificity/virulence in particular strains. There is also increasing evidence that avirulent strains of Y. ruckeri exist (30). In the present study, none of the Norwegian serotype O1 isolates cultured from the egg fluid of otherwise healthy brood stock salmon, or from biofilm within farming sites with no recorded history of yersiniosis, fell within the disease-associated CC 1 by MLVA. Instead, such isolates appeared entirely as singletons or formed distinct clonal complexes. In particular, CC 7 and CC 8 and 9, respectively, consist of nonclinical serotype O1 isolates recovered primarily from two separate yersiniosis-free freshwater salmon farms (Table 2). Conceivably, clonal expansion of host-adapted, virulent Y. ruckeri strains, from essentially environmental and/or commensal background populations, may have occurred independently in several salmon-producing countries and resulted in the observed geographic endemism.

In conclusion, this broad population study of Yersinia ruckeri substantially expands on the existing epizootiological history of this important fish pathogen and supports or verifies previous notions of host specificity, geographic endemism, and anthropogenic dissemination. Particularly, we verify by MLVA that yersiniosis in international rainbow trout farming is dominated almost entirely by a clonal strain of Y. ruckeri serotype O1 (CC 2) which appears to have been spread on separate occasions from North America to the United Kingdom and continental Europe, respectively. In contrast, we find that yersiniosis in international salmon farming is dominated mainly by geographically restricted and presumably native clones for which a recent common ancestry has not yet been clearly established. We show that a single, exclusively Norwegian, Y. ruckeri serotype O1 clone (CC 1) dominates the disease situation in Norwegian salmon farming. The MLVA assay further enables separation of putatively virulent and avirulent serotype O1 strains in Norway and indicates long-standing colonization of freshwater farms with specific Y. ruckeri strains. The scheme thus offers an extremely sensitive epizootiological tool that yields easily interpretable data. The entire procedure from agar plate to inclusion in an MST cluster analysis may be completed in less than a working day.

## MATERIALS AND METHODS

### Bacterial strains.

A total of 484 Y. ruckeri isolates, including reference strains of serotypes O1, O2, O5, O6, and O7 (7, 12), covering 19 species of fish/animals or environmental sources, 19 countries (from four continents), and 7 decades (1959 to 2017), were included in this study (see Table S1 in the supplemental material). Stock cultures, cryopreserved at −80°C, were revived on 5% bovine blood agar (BA) and incubated at 22°C for 1 to 2 days prior to further processing. All Norwegian isolates were serotyped by slide agglutination as previously described (31), using antisera raised against Y. ruckeri serotypes O1 (NCTC 12266), O2 (NCTC 12267), and O5 (NCTC 12268). Isolates previously serotyped in other laboratories were not re-serotyped in the present study. Biotyping of selected isolates was conducted as previously described (32). For PCR, genomic DNA was extracted by boiling bacterial cells from a single colony in 50 μl Milli-Q water for 7 min, followed by centrifugation and use of the supernatant as template.

### Identification of informative VNTR loci.

Y. ruckeri genome assemblies retrieved from NCBI GenBank and/or generated in-house in participating laboratories (unpublished), representing a broad range of serotypes and spatiotemporal origins, were subjected to analysis with Tandem Repeats Finder v4 (33) in combination with BLAST searches. Of over one hundred putatively repetitive loci identified, 10 variable loci (Table 1), confirmed by singleplex PCR and Sanger sequencing (not shown), were selected for further MLVA development. VNTR locus selection criteria were (i) ubiquitous occurrence in Y. ruckeri, (ii) repeat unit size uniformity, (iii) extensive inter-strain copy number variation, and (iv) sufficiently conserved flanking regions. While minor repeat sequence heterogeneity was accepted, 100% conservation of repeat unit size was set as a requirement to allow precise calling of repeat numbers by CE. In accordance with suggested guidelines (34), the selected VNTR loci were annotated according to their position (closest kbp) within the PacBio-generated and circularized genome of Y. ruckeri strain CSF007-82 (accession no. LN681231).

### Multiplex PCR and capillary electrophoresis (CE).

Two multiplex PCR assays (I and II) (Table 3) were established, each containing five primer pairs designed using MPprimer software (35) to provide an appropriate intra-assay amplicon size range. Forward primers (Applied Biosystems) were 5′ labeled with either of three fluorescent dyes (6-carboxyfluorescein [6FAM], VIC, or NED). For each assay, care was taken to avoid amplicon size overlap between loci labeled with identical dyes.

**TABLE 3:** Primer sequences, concentrations used in multiplex PCR, and sizes of amplified VNTR locus flank regions

VNTR locus	Multiplex assay	Primer sequence (5′→3′)		Primer concn (μM)	Amplified flank (bp)
		Forward^*a*^	Reverse		
YR2365	I	6FAM-CCTCGGAAACATAACTTATCGGAC	CCTCTGAAAGAGTACATCTCAGCAT	0.2	146
YR3168	I	VIC-ATCACGAATAAACTCTTGGGTGGA	CCTACCGCATATTCCTGGCTAAAT	0.1	95 (94)^*b*^
YR1524	I	VIC-TAATCCAGGCAGAATGGCAAAAAC	AAAATGTCTGTGATGGACAGTTGC	0.1	375
YR2276	I	NED-GTACGGATTGACTTGCATCCAAAA	GATAAATTAATCGGCCACAAGTGA	0.1	103
YR3750	I	NED-GAGACAAAGGATGCAGAGTACTGG	CTGATGCAATAATGACAAAGCCCA	0.2	315
YR1070	II	6FAM-GGTTATGTATTTTCAACAACCGCGA	TCCAACTCACCAATAACCCATCAA	0.2	171 (170)^*b*^
YR57	II	VIC-CTGAGCTTGTAGTGGTGTACTGAT	CAGCAATGATTTGAGCTGTAGCAA	0.1	82
YR940	II	VIC-ACCACAGCATAGTGTTATCCCAAA	TAAACTCAACTTGATCTGTGCCCT	0.2	305
YR1899	II	NED-ATCCCAAAACTATCCGGTGACAAT	CACCAAGGTAACCCTAGGCTAATA	0.2	87
YR2794	II	NED-TTGGAGCATGAAATGAGTTTTCCG	AACTCTTTGCCGTATTCGGTTTTC	0.1	419

a 6FAM, VIC, and NED are 5′ dye labels.

b A single-base-pair deletion was identified in the left flank of some isolates (see Results).

Multiplex PCR mixtures (i.e., two per isolate tested) contained 12.5 μl 2× Multiplex PCR master mix (Qiagen), 0.1 to 0.2 μM each appropriate primer pair (Table 3), 2 μl DNA template, and a volume of RNase-free water amounting to a total reaction volume of 25 μl. Subsequent PCRs involved, for both assays, (i) 5 min at 95°C (ii) 30 cycles of 0.5 min at 95°C, 1.5 min at 60°C, and 1 min at 72°C, and (iii) 60 min at 68°C, followed by cooling to 4°C indefinitely. PCR products were verified by gel electrophoresis and then diluted 1:10 (vol/vol) in Milli-Q water. From the diluted samples, 0.5 μl was added to 9 μl Hi-Di formamide (Applied Biosystems) and 0.5 μl GeneScan 600 LIZ dye size standard v2.0 (Applied Biosystems). Samples were denatured for 3 min at 95°C prior to CE on an Avant 3500xl Genetic Analyser (Applied Biosystems) utilizing POP-7 polymer (Applied Biosystems) and the following settings: 5-s injections at 1.6 kV (32 V/cm) and a 32-min run time at 15 kV (300 V/cm) and 60°C.

### VNTR fragment size calling and MLVA profiling.

Electrophoretic peaks were identified and size-called in GeneMapper 5 (Applied Biosystems). Differences in VNTR fragment sizes as called by CE and Sanger sequencing were identified, a phenomenon previously attributed to biased amplicon mobility patterns in CE machines (36, 37). These discrepancies were stable in relation to allele size, and size calls were subjected to locus-specific correction and converted to VNTR repeat counts according to the following formula (sizes in base pairs): VNTR repeat count = (CE size call × *s* + *i* − amplified flank size)/VNTR repeat size, where *s* and *i* represent the slope and intersection point, respectively, for individual VNTR loci identified by plotting accurate PCR fragment sizes (as determined by Sanger sequencing) against corresponding fragment sizes called by CE. The line-of-best-fit equation for each locus was identified by linear regression utilizing data from 15 to 19 strains displaying various alleles and representative for the size spans observed (see Fig. S2 in the supplemental material). Each isolate was thus assigned a ten-digit integer string (MLVA profile) representing the number of whole repeats identified at each VNTR locus. Absent CE peaks were assigned a repeat count of 0.

### Allelic diversity and statistical evaluation.

Based on the observed allelic diversity, the discriminatory capacity of the studied VNTR loci, both individually and in combination, was evaluated by calculating SID values (38). Possible linkage disequilibrium among the loci was investigated using LIAN version 3.7 (39), employing the Monte Carlo model with 10 000 iterations. Only single representatives of each MLVA profile were included for LIAN analysis.

### MLVA cluster analysis.

MLVA profiles for all 484 isolates were imported into BioNumerics v6.6 (Applied Maths NV, Sint-Martens-Latem, Belgium), and MST cluster analysis performed with default settings. In the resulting MST diagram, a cluster (clonal complex) partitioning threshold of ≤4/10 nonidentical loci was employed, and branches representing >5/10 nonidentical loci were hidden.

### VNTR stability.

Six Y. ruckeri isolates, representing various MLVA clonal complexes, were subjected to 40 serial passages at 1- to 2-day intervals to assess the *in vitro* stability of the VNTR loci. For each passage, single colonies were resown onto fresh BA plates and incubated at 22°C. MLVA profiles were obtained as described above from colonies harvested after 0, 10, 20, 30, and 40 passages. To assess the short-term *in vivo* stability of the VNTR loci, MLVA typing was also performed on 19 Y. ruckeri isolates recovered from 13 Atlantic salmon sampled simultaneously during an ongoing yersiniosis outbreak in a Norwegian freshwater facility. The longer-term environmental stability of the VNTR loci under industrial aquaculture production conditions was assessed by MLVA typing performed on putative “house strain” isolates associated with four freshwater production sites for Atlantic salmon smolts with a history of recurring yersiniosis. For each site, 4 to 33 isolates, recovered over a period of 2 to 6 years, were examined.

### Comparative resolution of MLST versus MLVA.

DNA sequences for the six previously published MLST loci (15) were extracted from available Y. ruckeri genome assemblies (NCBI GenBank or unpublished). Published MLST sequences (15, 16) were downloaded from www.pubmlst.org/yruckeri and NCBI GenBank, respectively. AT and ST designations were assigned in accordance with, or as a continuation of, previous MLST studies (15, 16). Necessary trimming of two loci (see Results) resulted in reclassification of several previously published AT/ST profiles.

AT profiles from 134 isolates (involving 35 ST) for which both MLST and MLVA information was available (see Table S1 in the supplemental material) were subsequently imported into BioNumerics v6.6. The epizootiological resolution of the two methods was then compared by MST cluster analyses (default settings) based on MLVA and MLST data, respectively. The truncated DNA sequences underlying the modified MLST were also subjected to MLSA. As such, the concatenated gene sequences were aligned with ClustalX (40) and used for constructing a maximum-likelihood tree in MEGA6 (41) with default settings employed.

### Accession number(s).

DNA sequences corresponding to the four novel Y. ruckeri MLST allele types identified during the current study were submitted to NCBI GenBank under accession no. MH156841 to MH156844 (see Table S3 in the supplemental material).

## Supplementary Material

Supplemental material
