# Supplementary material for: Multilocus Variable-Number Tandem-Repeat Analysis of Yersinia ruckeri Confirms the Existence of Host Specificity, Geographic Endemism, and Anthropogenic Dissemination of Virulent Clones
Source: Appl Environ Microbiol. 2018 Aug 1;84(16):e00730-18. doi: 10.1128/AEM.00730-18 (PMC6070765; doi:10.1128/AEM.00730-18)
Supplement: Supplemental material [file supp_84_16_e00730-18__index.html]

Supplemental material 

# Multilocus Variable-Number Tandem-Repeat Analysis of Yersinia ruckeri Confirms the Existence of Host Specificity, Geographic Endemism, and Anthropogenic Dissemination of Virulent Clones

## Supplemental material

- Supplemental file 1 -

  Metadata on all *Y. ruckeri* strains/isolates included in this study (Table S1).

  XLSX, 33K
- Supplemental file 2 -

  VNTR repeat counts (whole repeats) and MLVA clonal complex affiliations of *Y. ruckeri* strains/isolates investigated in this study (Table S2).

  XLSX, 39K
- Supplemental file 3 -

  MLST allele type and sequence type designations for *Y. ruckeri* strains/isolates investigated in this study (Table S3).

  XLSX, 17K
- Supplemental file 4 -

  Example electropherograms visualizing the ten VNTR amplified from a single *Y. ruckeri* isolate (Fig. S1); graphs showing locus-specific line-of-best-fit curves and associated equations used for correcting VNTR fragment sizes (Fig. S2); alignments visualizing inconsistencies observed in *thrA* and *recA* gene sequences as reported for identical *Y. ruckeri* strains (Fig. S3); minimum spanning trees based on MLVA and modified MLVA data (Fig. S4); maximum likelihood tree inferring phylogenetic distances within the examined *Y. ruckeri* MLST data set (Fig. S5).

  PDF, 1.2M
