# Supplementary material for: Multilocus Variable-Number Tandem-Repeat Analysis of Yersinia ruckeri Confirms the Existence of Host Specificity, Geographic Endemism, and Anthropogenic Dissemination of Virulent Clones
Source: Appl Environ Microbiol. 2018 Aug 1;84(16):e00730-18. doi: 10.1128/AEM.00730-18 (PMC6070765; doi:10.1128/AEM.00730-18)
Supplement: Supplemental material [file AEM.00730-18_zam016188666s4.pdf]

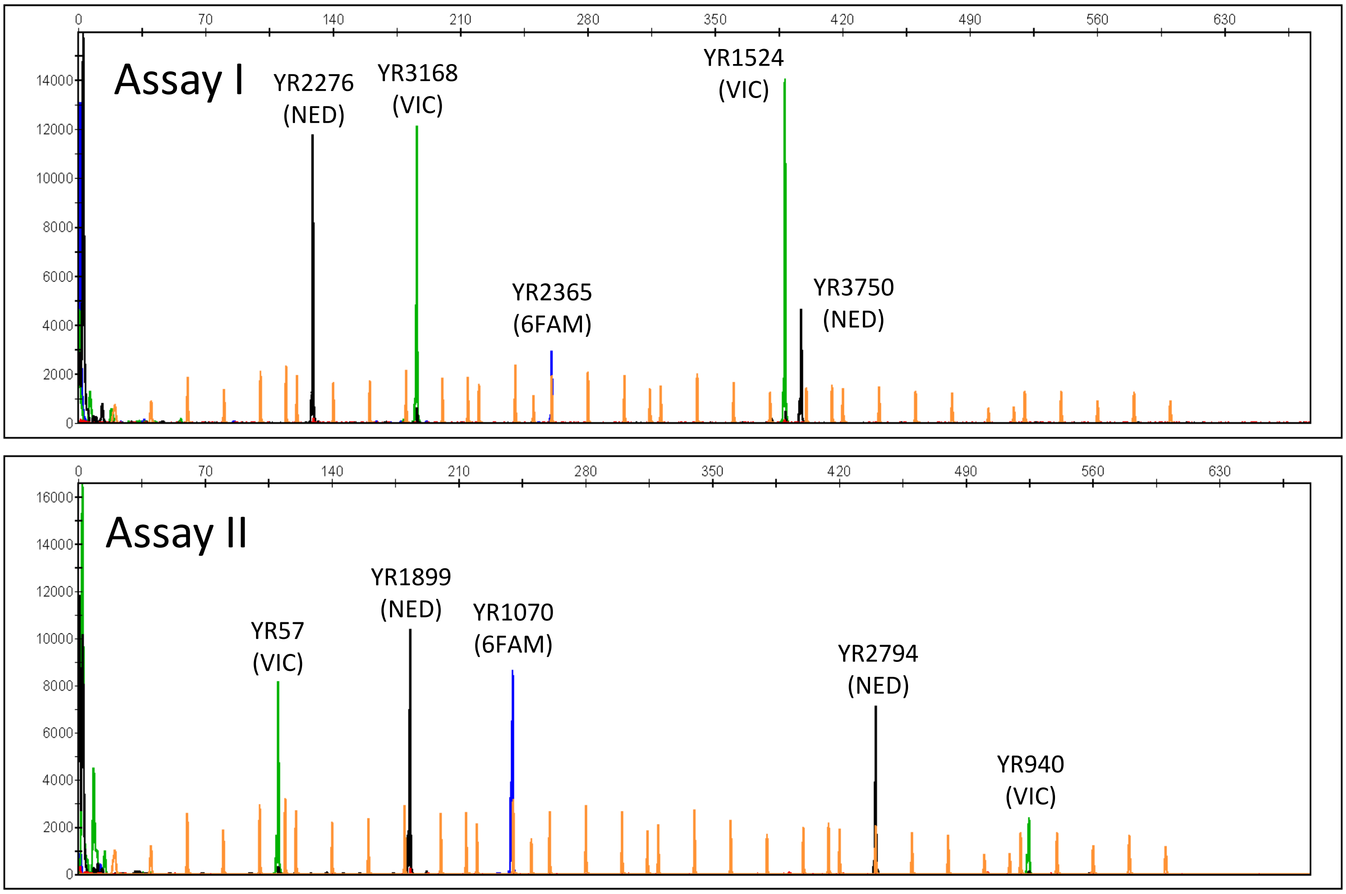

Figure S1: Example electropherograms visualising the ten VNTR loci amplified from a single *Y. ruckeri* isolate. The ten loci, distributed evenly amongst two multiplex PCR assays, were distinguished based on fluorescent labelling (indicated) and size. Orange peaks represent the size standard employed.

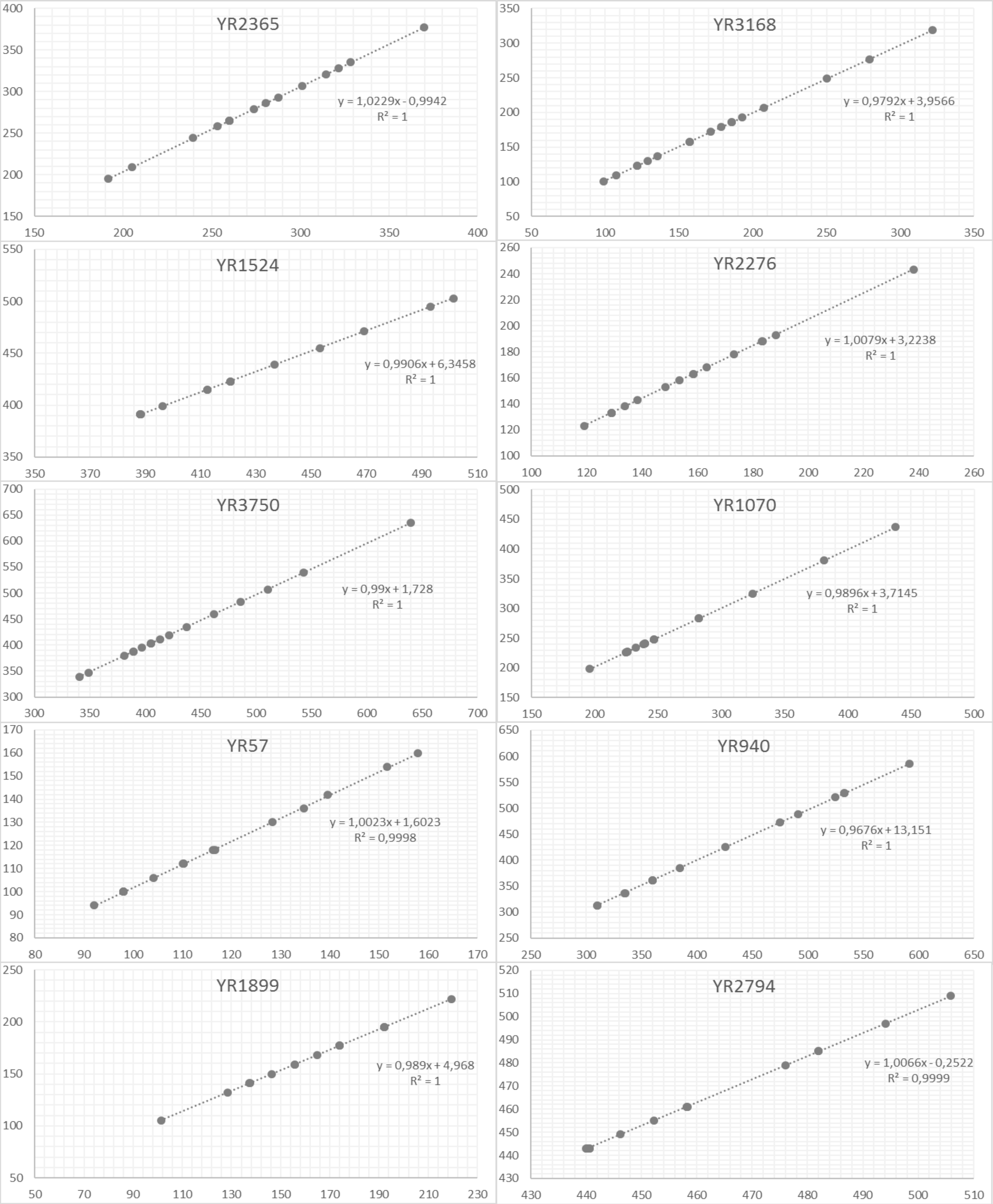

Figure S2: Graphs showing locus-specific line-of-best-fit curves and associated equations used for correcting VNTR fragments sizes called by capillary electrophoresis (CE). Linear regressions were based on comparison of VNTR fragment sizes as called by CE (x-axis) and Sanger sequencing (y-axis), respectively, from a collection of samples representative for the observed VNTR allele size ranges.

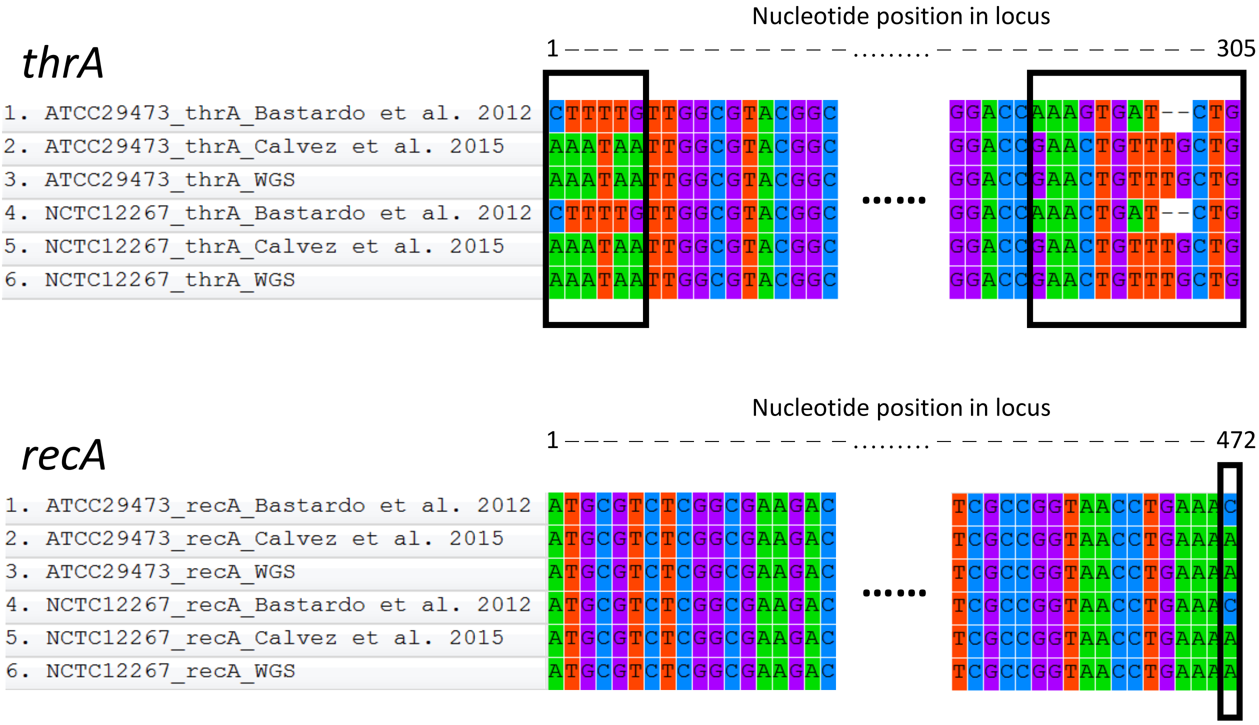

Figure S3: Alignments visualising inconsistencies (framed) observed in *thrA* (top) and *recA* (bottom) gene sequences as reported for identical *Y. ruckeri* strains in two different MLST studies (1, 2). While the alignments show only two reference strains (ATCC 29473 and NCTC 12267), the inconsistencies occurred throughout the respective datasets. Also shown are sequences extracted from the NCBI GenBank genomes (WGS) of these two strains, which are in agreement with the latter study (2).

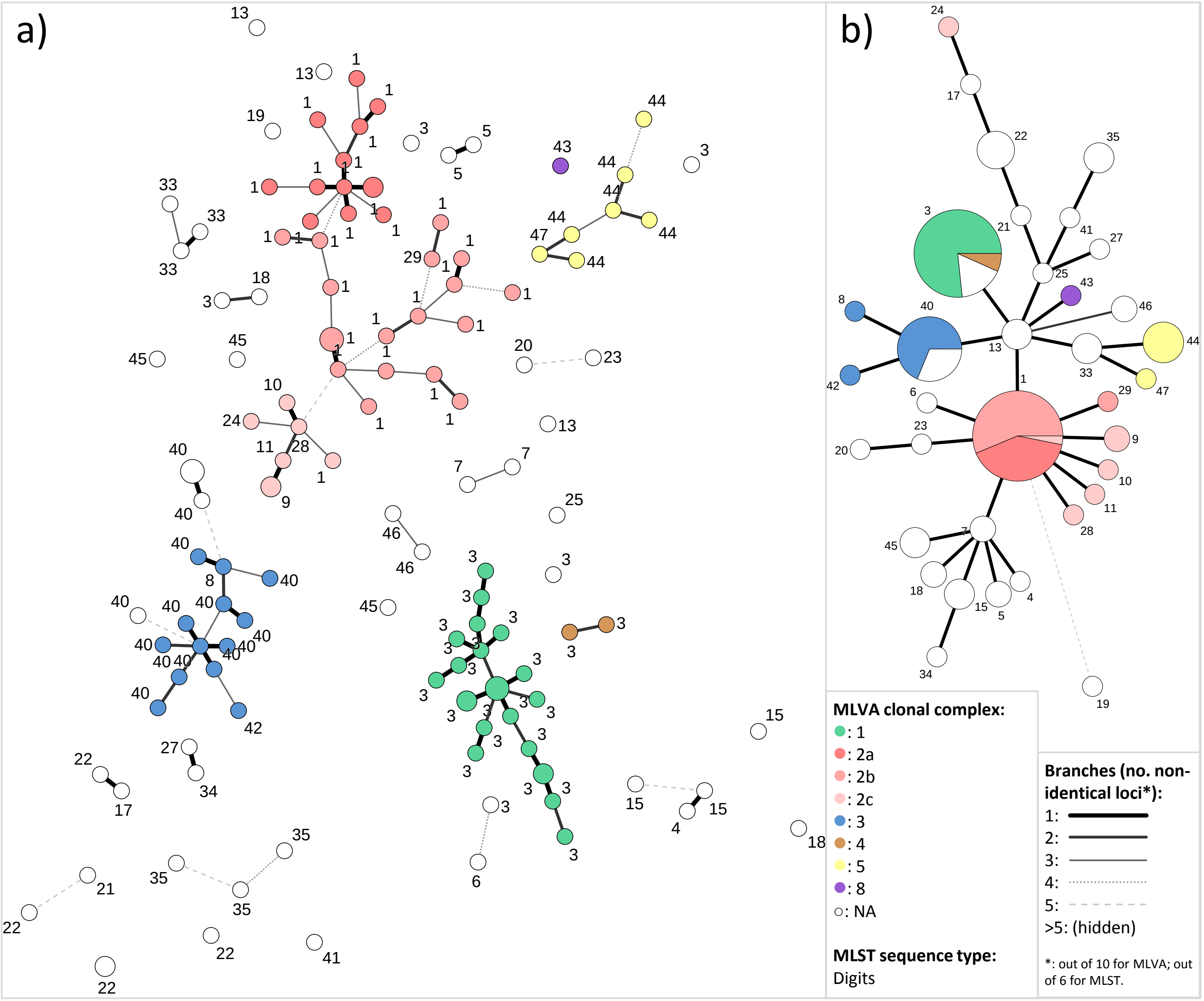

Figure S4: Minimum spanning trees based on MLVA (a) and modified (see Results) MLST (b) data (Tables S2 and S3, respectively), visualising the higher resolution awarded by MLVA. Only isolates typed by both methods are included. The affiliation of nodes with MLVA clonal complexes and MLST sequence types are indicated by colours and digits, respectively (see legend). Branch representations for declining MLVA and MLST similarity are shown (see legend).

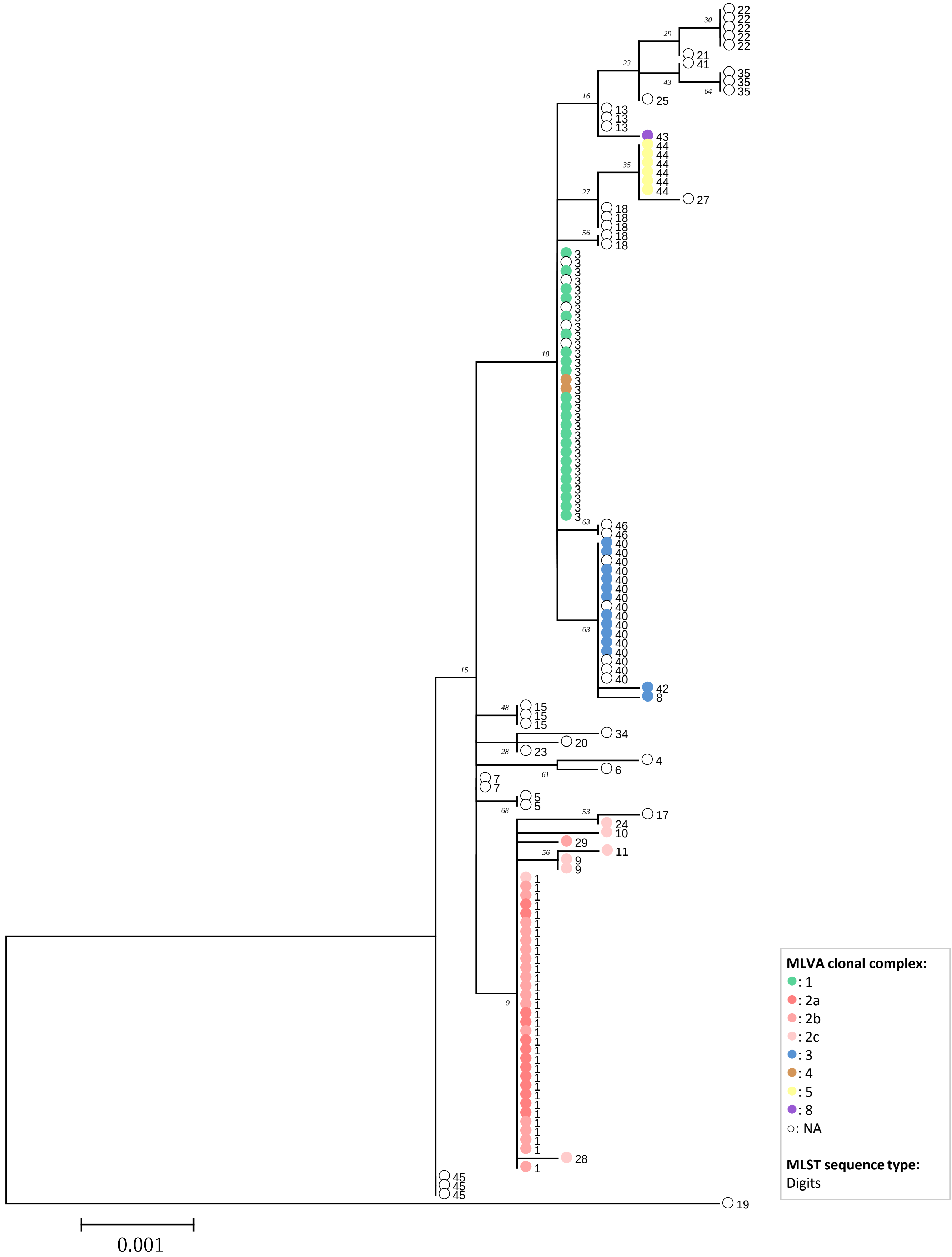

Figure S5: Maximum likelihood tree inferring, through MLSA, the phylogenetic distances within the examined *Y. ruckeri* MLST dataset (see Figure S4). Strain QMA0440 (ST 47), which lacks the *glnA* locus, was excluded from the alignment. The tree was created in MEGA6 employing default settings (500 bootstrap replications).

Supplementary references

1. Bastardo A, Ravelo C, Romalde JL. 2012. Multilocus sequence typing reveals high genetic diversity and epidemic population structure for the fish pathogen *Yersinia ruckeri*. Environ Microbiol 14:1888–1897.
2. Calvez S, Mangion C, Douet DG, Daniel P. 2015. Pulsed-field gel electrophoresis and multi locus sequence typing for characterizing genotype variability of *Yersinia ruckeri* isolated from farmed fish in France. Vet Res 46:73.
